# Effects of coronavirus disease 2019 lockdown on metabolic syndrome and its components among Chinese employees: A retrospective cohort study

**DOI:** 10.3389/fpubh.2022.885013

**Published:** 2022-08-05

**Authors:** Weiwei Xu, Yujuan Li, Yixin Yan, Liyun Zhang, Junhui Zhang, Chao Yang

**Affiliations:** ^1^Health Management Center, The Affiliated Hospital of Southwest Medical University, Luzhou, China; ^2^Department of Epidemiology and Health Statistics, School of Public Health, Southwest Medical University, Luzhou, China

**Keywords:** COVID-19, pandemic, metabolic syndrome, metabolic health, lockdown

## Abstract

**Objective:**

Coronavirus disease 2019 (COVID-19) and the accompanying isolation have changed resident life rhythms and behaviors. This study investigated the effects of the COVID-19 pandemic on metabolic syndrome (MetS) and its components in employees in southwestern China.

**Methods:**

This retrospective cohort study included 3,777 employees of five institutions who underwent physical examinations at the Affiliated Hospital of Southwest Medical University for three consecutive years from 2018 to 2020. We collected data on participant age and sex and measured the component indices of metabolic syndrome, including waist circumference, blood pressure (systolic and diastolic), fasting blood glucose level, and blood lipid (triglyceride and high-density lipoprotein cholesterol) level. We applied t-, chi-square, Mann–Whitney U, and Friedman's M tests to compare metabolic variables at different times.

**Results:**

The incidence of MetS in 2020 was 18.6%, significantly higher than the prevalence of 15.7% before the epidemic. The number of abnormal MetS components following the COVID-19 lockdown was much greater than those in 2018 (*P* < 0.001) and 2019 (*P* < 0.001), with no significant variations between the two years (*P* = 0.142*)*. All metabolic parameters, except for fasting blood glucose, were significantly worse than those pre-lockdown. The increase in the prevalence of MetS and all its abnormal components except for fasting glucose from 2019 to 2020 was significantly higher than that from 2018 to 2019. The change values between 2019–2020 and 2018–2019 for all indices except for diastolic blood pressure did not differ significantly between men and women. For all component indicators except for waist circumference, we observed no significant age differences in the growth differentials between the two periods (2019–2020 and 2018–2019).

**Conclusions:**

COVD-19 lockdown have increased metabolic health risks among Chinese adults. Targeted measures, such as health education, are urgently needed to address poor metabolic health caused by the COVID-19 pandemic.

## Introduction

Due to the current coronavirus disease 2019 (COVID-19) pandemic, governments in most affected nations have imposed tight confinement policies for their populations (1–3). These include working from home and closing schools, shops, restaurants, and any other non-essential businesses or services to halt the spread of the pandemic and thus avert the collapse of healthcare systems. Self-isolation at home due to lockdown unavoidably modifies inhabitants' life routines and, therefore, is associated with lower levels of physical activity, longer sedentary time, and dietary changes (4–7). The resulting reduction in daily energy consumption and increase in calorie intake may have unfavorable health consequences.

Metabolic syndrome (MetS), also known as insulin resistance syndrome, is a group of metabolic disorders that includes obesity, high blood pressure, dyslipidemia, and impaired glucose metabolism. MetS is strongly linked to increased risks of cardiovascular disease (CVD), type 2 diabetes mellitus (DM), and overall mortality (8, 9). The prevalence of MetS is relatively high, affecting 20–50% of the general population in most Western (10) and Asian (11) countries, and significantly increases with age (10). Before the COVID-19 pandemic, MetS was a major public health concern worldwide.

Many studies have assessed the relationship between epidemic isolation and weight gain (3, 4, 12–14); however, few have evaluated the impact of the COVID-19 pandemic on MetS and its components. Therefore, this study explored the impact of the COVID-19 lockdown on MetS and its components through longitudinal data over three consecutive years of 3,777 adults in southwestern China.

## Materials and Methods

### Study design and subjects

**Figure 1:**
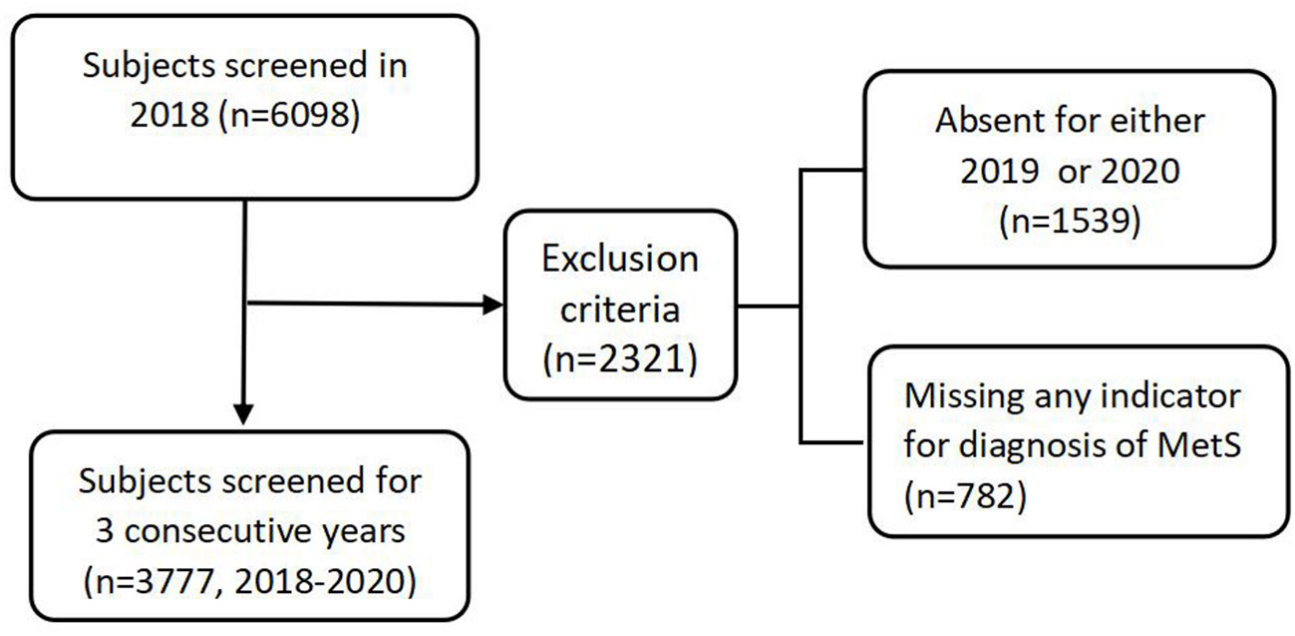
Flow diagram of subjects' selection.

We retrospectively extracted data from the physical examination system of the First Affiliated Hospital of Southwest Medical University (Luzhou, China). In the present study, all employees were invited by their companies to participate in annual medical checkups each year, and participation was based on the voluntary principle. A total of 6,098 individuals who met the following criteria were included in the study: (1) from five companies that had medical examinations at the hospital in 2018, and (2) were 18 years of age or older. Then, 2,321 participants were excluded for being absent from physical examination in 2019 or 2020 (1,539 subjects) or for missing blood biochemical indicators for MetS diagnosis (782 subjects). Thus, the present study included the remaining 3,777 individuals who participated in the checkup for three consecutive years (Figure 1). In 2020, the physical examinations were performed from April to December, immediately after the end of the lockdown. We utilized employees' metabolic indicators in 2019 and 2020 to represent the circumstances before and after the lockdown, respectively. To depict metabolic modifications in the year of the epidemic lockdown, we subtracted the component indices of MetS in 2019 from those in 2020. We also calculated the difference between 2019 and 2018 to determine the changes in indicators in a year without the COVID-19 pandemic.

### Measurements

The examinations included anthropometric measurements and biochemical tests. Systolic blood pressure (SBP) and diastolic blood pressure (DBP) were measured using an automated sphygmomanometer with the participant in a seated position. Waist circumference (WC) was measured in the horizontal plane halfway between the lowest border of the ribs and iliac crest to the nearest 0.5 cm. Standing height and body weight were measured without shoes or heavy clothing, and the body mass index (BMI) was reported in kg/m^2^. All examinations were performed between 8 AM and 11 AM after a 12-h overnight fast before the participants performed any exercise. Biochemical testing was performed using an automatic biochemical analyzer (SIEMENS ADVIA2400) to measure the levels of triglycerides (TG), high-density lipoprotein cholesterol (HDL-c), and fasting plasma glucose (FPG). In addition, data on age, sex, and previous hypertension or diabetes were collected.

### Definition of MetS

We used the 2009 harmonizing definition criteria of MetS^(16)^, as follows: central obesity: WC ≥ 85 cm in Asian men or WC ≥ 80 cm in Asian women; high blood pressure: SBP ≥130 mmHg, DBP ≥85 mmHg, or use of antihypertensive drugs; high TG: TG ≥150 mg/dL; low HDL-c: HDL-c <40 mg/dL in men or HDL-c <50 mg/dL in women; and hyperglycemia: FPG ≥100 mg/dL or use of antidiabetic agents. MetS was defined as the presence of three of these five components.

### Statistical analysis

Means with standard deviation (SD) or medians (25th−75th percentiles) were used to describe continuous variables, while numbers and percentages were used to express categorical variables. The MetS prevalence was calculated by dividing the number of people with abnormal components ≥3 by the total number of people. The 95% confidence intervals for prevalence were calculated using the formula p ± 1.96 $\sqrt{\frac{\text{p}\left(1-\text{p}\right)}{\text{n}}}$. Paired chi-square tests were used to compare the prevalence of MetS between the pre-lockdown and post-lockdown periods, while Friedman's M test was used to compare the number of abnormal MetS components among the 3 years. Multiple comparisons between any two years from 2018 to 2020 were made using the Wilcoxon signed-rank sum test and corrected for *p*-values using the Bonferroni method. Paired t- or Wilcoxon signed-rank tests were used to compare MetS component markers between 2019 and 2020 or to compare the metabolic modifications in the year of the pandemic lockdown with those in a normal year.

Stratified analysis was conducted according to age and sex in each subgroup. In China, 60 years old is not only the dividing line between middle age and old age, but also the last retirement age for most people. People under and over 60 years of age have different daily routines and therefore may be affected differently by the COVID-19 lockdown. Thus, we divided the subjects into two groups by 60 years of age and compared them in the present study. Comparison of metabolic indices between different subgroups was performed using two independent sample t-tests or Mann–Whitney U tests. All statistical tests were two-sided, with *p* < 0.05 considered significant. All statistical analyses were conducted using IBM SPSS version 25.0 (IBM Corp., Armonk, NY, USA).

## Results

**Figure 2:**
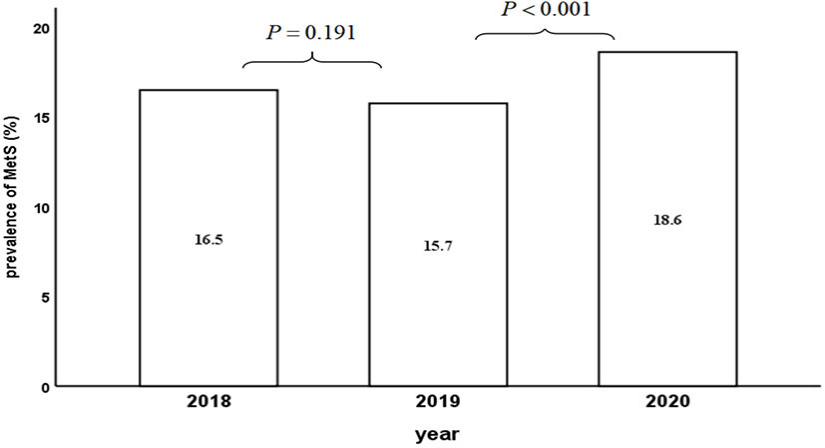
Three-consecutive-year MetS prevalence of 3777 individuals.

Of the 3,777 subjects, 2,183 were male (57.8%), with an average age of 44.9 ± 12.9 years, and 1070 (28.3%) were obese in 2018. Of the 2,321 individuals excluded, 1,347 (58.0%) were male, with a mean age of 44.4 ± 15.8 years. Both the age and gender composition difference between the two populations were not statistically significant. Except for the slightly higher SBP and FPG in the excluded group, the differences between the two groups in the remaining indicators were not statistically significant (Table 1). The prevalence of MetS following the COVID-19 lockdown was 18.6% (95%CI: 17.3% - 19.8%), which was substantially higher than the prevalence pre-lockdown (15.7% in 2019, 95%CI: 14.6–16.9%), despite no notable differences between the two years (2018 and 2019) before the pandemic (Figure 2).

**Table 1:** Comparison of characteristics between included and excluded subjects at baseline.

**Variable**	**Total**	**Included**	**Excluded**	***P***
	***n* = 6,098**	***n =* 3,777**	***n =* 2,321**	
Age	44.7 ± 14.1	44.9 ± 12.9	44.4 ± 15.8	0.198
Sex (male/female)	3,530/2,568	2,183/1,594	1,347/974	0.855
SBP	125.9 ± 18.2	125.5 ± 17.9	126.7 ± 18.8	0.013
DBP	77.4 ± 11.7	77.4 ± 11.5	77.4 ± 12.0	0.968
BMI	23.3 ± 3.3	23.3 ± 3.1	23.2 ± 3.5	0.482
WC	79.5 ± 12.5	79.3 ± 13.6	79.9 ± 10.1	0.052
TG	1.33 (0.94, 2.01)	1.33 (0.95, 2.03)	1.33 (0.94, 2.01)	0.595
HDL-c	1.33 ± 0.34	1.33 ± 0.34	1.33 ± 0.34	0.514
FPG	5.19 ± 1.41	5.04 ± 1.04	5.44 ± 1.85	<0.001

BMI, body mass index; WC, waist circumference; SBP, systolic blood pressure; DBP, diastolic blood pressure; TG, triglycerides; HDL-c, high-density lipoprotein cholesterol; FPG, fasting plasma glucose.

Data are expressed as mean ± SD or median (25th to 75th percentiles). Independent t- or Wilcoxon rank tests were used to compare the indexes between included subjects and excluded subjects.

The difference in the number of abnormal MetS components in this cohort from 2018 to 2020 was statistically significant (*P* < 0.001). Multiple comparisons revealed a significantly higher number of abnormal MetS components following the COVID-19 lockdown compared to those in 2018 and 2019 (both *P* < 0.001), with no significant difference between the 2 years (*P* = 0.142) (Table 2).

**Table 2:** Numbers of abnormal MetS components from 2018 to 2020.

**Year**	**Number of MetS components**						**Mean rank**	***P-value***		
	**0**	**1**	**2**	**3**	**4**	**5**		**2018 vs. 2019**	**2018 vs. 2020**	**2019 vs. 2020**
2018	1,342 (35.5)	1,066 (28.2)	747 (19.8)	420 (11.1)	174 (4.7)	28 (0.7)	1.98	0.142	<0.001	<0.001
2019	1,400 (37.1)	1,046 (27.7)	737 (19.5)	405 (10.7)	161 (4.3)	28 (0.7)	1.94			
2020	1,247 (33.0)	1,051 (27.8)	777 (20.6)	459 (12.2)	201 (5.3)	42 (1.1)	2.08			

MetS: metabolic syndrome.

Data are expressed as numbers and percentages. Friedman's M test was used to compare the number of MetS components among the 3 years.

P-value: adjusted by Bonferroni correction for multiple tests.

Compared to the pre-lockdown rate in 2019, the anomaly rate of all five MetS components increased significantly after the lockdown. In contrast, only central obesity prevalence increased significantly from 2018 to 2019 (*P* = 0.010), while the anomaly rate of the other four components (high blood pressure, high TG, low HDL-c, and high FBG) decreased (Table 3). Moreover, the increased central obesity rate before and after the COVID-19 lockdown was 5.3%, significantly higher than that between 2018 and 2019 (1.6 %) (*P* < 0.001).

**Table 3:** The impact of the COVID-19 lockdown on abnormal rates of five MetS components.

**Variable**	**2018**	**2019**	**2020**	**2019–2018**	**2020–2019**	***P*_1_**	***P*_2_**
Central obesity (*n*, %)	822 (21.8)	883 (23.4)	1,085 (28.7)	61 (1.6)	202 (5.3)	0.010	<0.001
High blood pressure (n, %)	1,113 (29.5)	1,068 (28.3)	1,188 (31.5)	−45 (−1.2)	120 (3.2)	0.066	<0.001
High TG (*n*, %)	1,268 (33.6)	1,155 (30.6)	1,209 (32.0)	−113 (−3.0)	54 (1.4)	<0.001	0.031
Low HDL-c (*n*, %)	993 (26.3)	969 (25.7)	1,015 (26.9)	−24 (−0.6)	22 (0.6)	0.322	<0.001
High FBG (*n*, %)	460 (12.2)	444 (11.8)	499 (13.2)	−16 (−0.4)	55 (1.5)	0.403	0.002

Data are expressed as numbers and percentages. Paired chi-square tests were used to compare the rates at two different time points.

COVID-19, coronavirus disease 2019; MetS, metabolic syndrome; triglyceride; HDL-c, high-density lipoprotein cholesterol; FPG, fasting plasma glucose.

Central obesity was defined as waist circumference (WC)≥ 85 cm in men or ≥80 cm in women. High blood pressure: systolic blood pressure (SBP) ≥130 mmHg, diastolic blood pressure (DBP) ≥85 mmHg, or use of antihypertensive drugs. High TG: TG level ≥150 mg/dl. Low HDL-c: HDL-C <40 mg/dL in men or HDL-C <50 mg/dL in women. High FPG: FPG ≥100 mg/dL or use of antidiabetic agents.

2019–2018: abnormal rate change from 2018 to 2019.

2020–2019: abnormal rate change from 2019 to 2020.

P_1_: P-value of the comparison between 2018 and 2019.

P_2_: P-value of the comparison between 2019 and 2020.

We found similar results in our analysis of the component indices of MetS. Compared to 2019, all MetS component metrics, except for FPG, were adversely altered in 2020. Furthermore, the change during the pandemic lockdown (2019–2020) differed significantly from that between 2018 and 2019 (Table 4).

**Table 4:** The impact of the COVID-19 lockdown on MetS component indicators.

**Variable**	**2018**	**2019**	**2020**	**2019–2018**	**2020–2019**	***P*_1_**	***P*_2_**
WC	79.1 ± 9.5	79.7 ± 9.5	81.2 ± 9.4	0.6 ± 5.2	1.6 ± 5.4	<0.001	<0.001
SBP	118.4 ± 17.9	117.8 ± 18.1	118.8 ± 16.7	−0.6 ± 13.2	1.0 ± 12.2	<0.001	<0.001
DBP	71.4 ± 11.5	71.4 ± 11.6	71.8 ± 10.5	−1.9 ± 9.1	0.3 ± 8.5	0.036	<0.001
TG	1.33(0.95,2.03)	1.25(0.90,1.88)	1.30(0.89,1.97)	−0.08(−0.38,0.20)	0.04(−0.23,0.32)	<0.001	<0.001
HDL-c	1.33 ± 0.34	1.34 ± 0.34	1.33 ± 0.34	0.01 ± 0.18	−0.01 ± 0.17	0.010	0.001
FPG	5.04 ± 1.04	5.03 ± 0.92	5.02 ± 0.93	−0.01 ± 0.69	−0.01 ± 0.58	0.225	0.813

COVID-2019, coronavirus disease 2019; WC, waist circumference; SBP, systolic blood pressure; DBP, diastolic blood pressure; TG, triglycerides; HDL-c, high-density lipoprotein cholesterol; FPG, fasting plasma glucose.

Data are expressed as mean ± SD or median (25th to 75th percentiles). Paired t- or Wilcoxon signed-rank tests were used to compare the indexes at different time points or periods.

2019–2018: increase in indicators from 2018 to 2019.

2020–2019: increase in indicators from 2019 to 2020.

P_1_: P-value of the comparison between 2019 and 2020.

P_2_: P-value of the comparison between 2020–2019 and 2019–2018.

Four indicators (WC, SBP, DBP, and TG) in men and five indicators (WC, SBP, DBP, TG, and HDL-c) in women showed significantly higher unfavorable changes between 2019 and 2020 than those between 2018 and 2019. However, for all component indicators except FPG, we observed no significant sex differences in the growth differentials between the two periods (2019–2020 and 2018–2019) (Table 5).

**Table 5:** The impact of COVID-19 lockdown on MetS component indicators in different sexes.

**Variable**	**Male (*****n =*** **2,183)**			**Female(*****n =*** **1,594)**			***P1***	***P2***	***P3***
	**2019–2018**	**2020–2019**	***d***	**2019–2018**	**2020–2019**	***d***			
WC	0.7 ± 5.1	1.5 ± 5.4	0.9 ± 8.6	0.4 ± 5.2	1.6 ± 5.4	1.2 ± 9.1	<0.001	<0.001	0.963
SBP	−1.4 ± 13.9	0.2 ± 13.1	1.6 ± 23.1	0.4 ± 12.2	2.2 ± 10.8	1.8 ± 19.4	0.001	<0.001	0.784
DBP	−2.2 ± 9.4	−0.5 ± 8.7	1.6 ± 15.5	−1.5 ± 8.8	1.4 ± 8.0	3.0 ± 14.4	<0.001	<0.001	0.006
TG	−0.09 (−0.44,0.22)	0.04 (−0.27,0.37)	0.17 (−0.36,0.78)	−0.06 (−0.32,0.17)	0.03 (−0.19,0.26)	0.14 (−0.26,0.54)	<0.001	<0.001	0.210
HDL-c	0.00 ± 0.18	−0.01 ± 0.16	−0.01 ± 0.28	0.01 ± 0.18	−0.01 ± 0.19	−0.02 ± 0.31	0.074	0.005	0.282
FPG	0.00 ± 0.73	−0.01 ± 0.64	−0.01 ± 1.15	−0.02 ± 0.62	−0.02 ± 0.50	0.00 ± 0.97	0.709	0.910	0.730

Data are expressed as mean ± SD or median (25th to 75th percentiles). The paired t-test or Wilcoxon signed rank test was used to compare the indexes at two different time points or periods. An independent t-test or Mann-Whitney U test was used to compare the indexes between sexes.

WC, waist circumference; SBP, systolic blood pressure; DBP, diastolic blood pressure; TG, triglyceride; HDL-c, high-density lipoprotein cholesterol; FPG, fasting plasma glucose.

2019-2018: the add value of indicators from 2018 to 2019. 2020-2019: the add value of indicators from 2019 to 2020.

d: Difference between the added value from 2019 to 2020 and from 2018 to 2019.

P1: P value of comparison between 2020-2019 and 2019-2018 in men.

P2: P value of comparison between 2020-2019 and 2019-2018 in women.

P3: the P value of d comparison between men and women.

Four indicators (WC, SBP, DBP, and TG) in the <60-years group and two indicators (TG and HDL-c) in the ≥60-years group showed significantly higher unfavorable changes between 2019 and 2020 than those between 2018 and 2019. However, for all component indicators except for WC, there were no significant age differences in growth differentials between the two periods (2019–2020 and 2018–2019) (Table 6).

**Table 6:** The impact of the COVID-19 lockdown on MetS components indicators in different age groups.

**Variable**	<**60 years (*****n =*** **3,277)**			≥**60 years (*****n =*** **500)**			***P*_1_**	***P*_2_**	***P*_3_**
	**2019–2018**	**2020–2019**	***d***	**2019–2018**	**2020–2019**	***d***			
WC	0.6 ± 5.1	1.6 ± 5.4	1.2 ± 8.7	0.6 ± 5.2	0.5 ± 5.6	−0.1 ± 9.0	<0.001	0.812	0.003
SBP	−0.6 ± 13.0	1.1 ± 11.7	1.8 ± 21.0	−0.7 ± 14.9	0.4 ± 15.2	1.1 ± 25.6	<0.001	0.335	0.573
DBP	−1.9 ± 9.1	0.5 ± 8.2	2.3 ± 14.7	−2.1 ± 9.4	−0.8 ± 9.8	−1.3 ± 16.8	<0.001	0.088	0.191
TG	−0.08 (−0.38,0.20)	0.04 (−0.22,0.32)	0.16 (−031,0.66)	−0.05 (−0.37,0.18)	−0.01 (−0.26,0.26)	0.09 (−0.37,0.62)	<0.001	0.001	0.158
HDL-c	0.01 ± 0.17	0.00 ± 0.17	−0.01 ± 0.29	0.01 ± 0.22	−0.03 ± 0.17	−0.04 ± 0.32	0.019	0.008	0.062
FPG	0.00 ± 0.66	−0.01 ± 0.53	−0.02 ± 1.01	−0.08 ± 0.84	0.00 ± 0.85	0.08 ± 1.42	0.350	0.224	0.155

COVID-2019, coronavirus disease 2019; WC, waist circumference; SBP, systolic blood pressure; DBP, diastolic blood pressure; TG, triglycerides; HDL-c, high-density lipoprotein cholesterol; FPG, fasting plasma glucose.

Data are expressed as mean ± SD or median (25th to 75th percentiles). Paired t or Wilcoxon signed-rank tests were used to compare indexes at different time points or periods. Independent t or Mann–Whitney U tests were used to compare indexes between sexes.

2019–2018: sum of indicators from 2018 to 2019.

2020–2019: sum of indicators from 2019 to 2020.

d: Difference between the sum from 2019 to 2020 and 2018 to 2019.

P_1_: P-value of the comparison between 2020–2019 and 2019–2018 in the <60-years group.

P_2_: P-value of the comparison between 20202019 and 20192018 in the ≥60-years group.

P_3_: P-value of d comparison between two age groups.

## Discussion

Since the outbreak of the COVID-19 pandemic, few studies have focused on the association between epidemic closure and the metabolic health of the population. A study in Italy reported a significantly higher prevalence of obesity, dyslipidemia, and MetS in the study subjects following the lockdown compared to that before the lockdown (15). However, this study included patients with endocrine disorders; moreover, the sample size was small and, therefore, not sufficiently representative. However, a study of 32,399 diabetic patients in Germany observed no evidence of harmful effects of the pandemic lockdown on the heart health of patients with type 2 diabetes (16). In the present study, the metabolic health status of Chinese adults after the pandemic lockdown was notably worse than that before the lockdown. This finding was supported not only by the increased prevalence of MetS and its component abnormalities but also by the rise of abnormal counts of MetS components and unfavorable changes in each metabolic indicator. Moreover, the unfavorable changes in all metabolic outcomes in the year after the COVID-19 pandemic (2019–2020) were also higher than those in the year before the pandemic lockdown (2018–2019). This trend remained robust after population stratification by sex and age. This evidence indicates that the COVID-19 pandemic lockdown increased the metabolic health risk of the general adult population in China.

The source of the weight gain induced by epidemic lockdown is also likely the root of its relationship with MetS. A recent international online survey in Asian, African, and European populations demonstrated the negative effects of COVID-19 home confinement on all physical activity intensity levels and that daily sitting time increased from 5 to 8 h per day, along with unhealthy food consumption and meal planning (5). A study in Korea also reported that social distancing resulted in irregular living patterns as well as decreased physical activity, which increased the risk of lifestyle-related diseases such as obesity and MetS (17). Therefore, the Korean Society for the Study of Obesity advocated maintaining or increasing levels of regular physical activity, adopting regular and balanced diets, and regular medical visits for people with chronic conditions to prevent MetS during the COVID-19 pandemic (17). Health education is a routine tool used in chronic disease management. A 3-year follow-up visceral fat study in Japan reported that a health education program reduced MetS risk (18). In the post-pandemic period, health education may play an essential role in addressing the metabolic health crisis caused by COVID-19.

### Limitation

The present study had several limitations. Due to practical constraints, we were unable to measure the participants at two precise time points before and after the pandemic lockdown but rather used data from their annual physical examinations. Thus, the changes in metabolic indicators may have been related not only to lockdown but also to other annual effects. In addition, with increasing age (by 1 year), the metabolic health indicators of the study participants may also change adversely. To overcome these shortcomings, we included indicators of the study population in 2018, and calculated the changes in metabolic indicators that occurred from 2018 to 2019, a year without a pandemic lockdown, as a control. The comparison of the difference in the change in metabolic indicators between the two periods (2018–2019 vs. 2019–2020) allowed our derivation of the possible impact on metabolic health due to the lockdown. Kowall et al. conducted a similar study in German patients with diabetes (16). Moreover, the gender composition of the included subjects and the proportion of individuals with metabolic diseases at baseline may affect the study results to some extent. Although our stratified analysis of age and gender yielded consistent results, selection bias could not be completely ruled out.

## Conclusion

COVID-19 lockdown have increased metabolic health risks among Chinese adults. Further programs like health education are urgently needed to address the metabolic health crisis caused by the COVID-19 pandemic.

## Data availability statement

The raw data supporting the conclusions of this article will be made available by the authors, without undue reservation.

## Ethics statement

The studies involving human participants were reviewed and approved by Ethics Committee of the First Affiliated Hospital (Clinical Medicine College, Southwest Medical University). The patients/participants provided their written informed consent to participate in this study.

## Author contributions

WX, CY, and YL contributed to conception and design of the study. YL organized the database. CY performed the statistical analysis. WX wrote the first draft of the manuscript. WX, YY, LZ, and JZ wrote sections of the manuscript. All authors contributed to manuscript revision, read, and approved the submitted version.

## Funding

This manuscript was supported by the Luzhou Municipal People's Government-Southwest Medical University Strategic Cooperation Program (2019LZ-XNYD-R01).

## Conflict of interest

The authors declare that the research was conducted in the absence of any commercial or financial relationships that could be construed as a potential conflict of interest.

## Publisher's note

All claims expressed in this article are solely those of the authors and do not necessarily represent those of their affiliated organizations, or those of the publisher, the editors and the reviewers. Any product that may be evaluated in this article, or claim that may be made by its manufacturer, is not guaranteed or endorsed by the publisher.
